# Adult outdoor group sport play during a pandemic: Feasibility, acceptability, and program adherence results from a study of modifications to mitigate COVID-19 risk

**DOI:** 10.1016/j.pmedr.2021.101476

**Published:** 2021-06-27

**Authors:** Matthew A. Ladwig, Christopher N. Sciamanna, Kayla N. Rutt, Joshua M. Blaker, Kalen Kearcher, Brandon J. Auer, Liza S. Rovniak, David E. Conroy, Jinger S. Gottschall, Matthew L. Silvis, Joshua M. Smyth, Ming Wang

**Affiliations:** a Penn State College of Medicine, Hershey, PA, USA; b Penn State University, University Park, PA, USA; c Les Mills International Ltd., New York, NY, USA

**Keywords:** COVID-19, Pandemic, Physical activity, Safety, Adherence, Enjoyment, Adults

## Abstract

The purpose of this study was to investigate the feasibility, acceptability, and short-term adherence to an adult outdoor group sport play program designed to maximize enjoyment that was modified to incorporate COVID-19 risk mitigation strategies. We enrolled 17 healthy, but sedentary, central Pennsylvania adults (*mean*_age_ = 31.6 ± 7.3) without obesity via mail advertisements to participate in four, 60-minute sessions of instructor-led modified sport games (e.g., handball, Ultimate Frisbee, soccer) over a two-week period in October 2020. Durign recrutiment and the study period, there were approximately 1000–2000 new cases of COVID-19 daily in central Pennsylvania . The overall adherence rate to the sessions was 91%, and no participants reported new or existing COVID-19 symptoms or diagnoses during the two-week study period or 10-days following the final session. Despite enforcing mandatory COVID-19 safety measures (e.g., universal masking, social distancing), the participants reported enjoying the program and feeling socially connected with one another, while perceiving the program to be safe. Additionally, all participants expressed a desire to participate again, if the program were offered in the future. These results suggest that an outdoor group sport play program for adults can be feasible, acceptable, and well-adhered to despite enforcing mandatory COVID-19 safety precautions. Physical activity programs similar to the one presented here may provide an easily-adaptable approach to outdoor physical activity during the current and potential future pandemics that have viral transmission characteristics similar to COVID-19.

## Highlights

• Attendance to an outdoor sport play program was high, despite COVID-19 precautions.
• Masking did not cause notable discomfort or problems socializing among participants.
• No new COVID symptoms/diagnoses were reported during or 10-days after 2-week study.
• All participants desired to continue program participation, if held in the future.

## 1 Introduction

The coronavirus disease 2019 (COVID-19) pandemic has caused major disruptions in the daily lives of millions of people in the United States and billions around the world. One highly apparent disruption to health-related behavior due to the pandemic is a reduction in physical activity (PA), driven by factors such as mandatory stay-at-home orders, quarantines, and fearfulness of contracting and/or transmitting the disease. Several large-scale studies have reported decreased PA along with increased sitting and screen time, especially among those who were most physically active prior to the pandemic (Pepin et al., 2020, Tison et al., 2020, Stockwell et al., 2021). The restrictions to daily life associated with the COVID-19 pandemic have rendered it difficult for many previously physically active individuals to continue their preferred mode of activity (e.g., indoor group fitness classes) and erected new roadblocks for those wishing to become more physically active. Moreover, in areas with the most restrictive stay-at-home orders (i.e., those restricting all but "essential" outdoor activities), individuals may be limited only to PA that can be done in the often space-limited confines of the home. The pandemic has also led to further decreased PA among already sedentary individuals with preexisting conditions and comorbidities such as obesity and type 2 diabetes (Ruiz-Roso et al., 2020) – at risk populations whose health-status can benefit from regular PA.

Other recent data suggest that the mental health of many people around the world has deteriorated over the course of the pandemic. Specifically, people report feeling less connected to others, more stressed, and more likely to report symptoms of depression and anxiety (Salari et al., 2020, Luo et al., 2020). Given the reciprocal relationships between PA and mental health (Stults-Kolehmainen and Sinha, 2014, Firth et al., 2016, Schuch et al., 2017, Schuch et al., 2016), deteriorations in either during a pandemic may lead to a vicious circle where one potentiates the deleterious effects of the other. Given the risks to mental and physical health posed by physical inactivity, researchers recommend that “public health strategies should include the creation and implementation of interventions that promote safe physical activity and reduce sedentary behaviour should other lockdowns occur (Stockwell et al., 2021).”

The most recent evidence suggests COVID-19 is an airborne virus transmitted from person-to-person through respiratory droplets released into the air when talking, coughing, or sneezing (Salian et al., 2021). When people spend prolonged periods of time in close contact with infected others, especially in poorly ventilated indoor areas, they may inhale larger quantities of respiratory droplets infected with COVID-19. This high viral load not only increases the probability of contracting the virus, but, as with SARS-CoV-1 (Crisp et al., 2012), is also associated with disease severity and mortality (Fajnzylber et al., 2020). However, interacting with others outdoors may present less risk, as fresh air can help to disperse the infected respiratory droplets (World Health Organization, 2009, American Society of Heating Refrigerating and Air-Conditioning Engineers et al., 2008). In this context, people may be less likely to inhale the quantities of respiratory droplets necessary to become infected with the virus, and, if they do, experience less severe symptoms.

In the rapidly changing landscape of the pandemic, transmission prevention is paramount to decrease risks to individuals in those communities most likely to experience severe illness symptoms or death (e.g., the elderly, those with preexisting conditions). These transmission prevention efforts are also crucial for athletes and physically active individuals to minimize interruptions in training and possible short-and long-term adverse effects on an infected individual’s respiratory and cardiovascular systems, such as myocarditis (Kim et al., 2021, Starekova et al., 2019). Spread of COVID-19 through respiratory droplets from coughing and sneezing as well as to a lesser degree by touching the eyes, nose, or mouth after touching surfaces containing infected respiratory droplets is of particular concern in athletics where players come in frequent close contact with one another. However, evidence suggests that, if transmission in the surrounding community is low and adequate risk mitigation is in place, the risk of COVID-19 to an individual athlete can be low (Carmody et al., 2020). Sport-specific protocols have been tailored to individual sports and are readily available online (Sikka et al., 2021, League, 2020, League, 2020, Aschburner, 2020, Institute, 2020, Pennsylvania Interscholastic Athletic Association, 2020) and are summarized in Table 1.

**Table 1:** Summary of COVID-19 safety protocols recommended by various sporting agencies (Sikka et al., 2021, League, 2020, League, 2020, Aschburner, 2020, Institute, 2020, Pennsylvania Interscholastic Athletic Association, 2020).

**COVID-19 safety recommendations for athletics**
Daily personal health assessments
Universal masking
Frequent sanitization of sport implements and equipment
Frequent hand hygiene
Avoid sharing personal items

Despite the accumulating evidence of how to safely “return to sport” during the COVID-19 pandemic, it is unclear how the PA experience of individuals may be effected by mandatory safety protocols or whether people actually have interest in returning to group PA during the pandemic. For example, recent evidence has shown that masking during incremental PA can lead to mild hypercapnea (Law et al., 2021). For some, similar responses during moderate-to vigorous-intensity sport and PA could lead to increased displeasure. With these questions in mind, our study team raised several concerns that led to the conception of the present study. These included: 1) whether individuals would enroll in the study at levels similar to pre-pandemic studies of our team, 2) would they participate if required to adhere to COVID-19 safety protocols, and 3) whether the masking and safety protocols impact their enjoyment and feelings of connectedness with others during the PA experience. COVID-19 will likely remain an international health concern for years to come and the goal is to return to safe, full-scale PA research with human participants . Therfore, the aim of this study was to understand whether an adult outdoor group sport play program designed to maximize enjoyment was feasible, acceptable, and would be adhered to by participants even with restrictive protocols in place to mitigate the transmission of COVID-19. Because of the unprecedented nature of the COVID-19 pandemic and unknowns associated with group PA during a pandemic (e.g., whether masking would be perceived as unpleasant or socialization would prove more difficult), we did not enter this short-term pilot with specific *a priori* hypotheses.

## 2 Methods

This study is part of a larger parent randomized-controlled trial of adult PA funded by the National Heart, Lung, and Blood Institute that was indefinitely suspended with the onset of the coronavirus pandemic, in March 2020 (Ladwig et al., 2021). The participants in the present study were not enrolled in the parent trial, but were a convenience sample recruited from the local community to pilot test COVID-19 risk mitigation strategies that could be incorporated into the parent trial, if still mandatory, once restrictions on full-scale human participant research were lifted. The present study took place during October 2020. To provide some context on the local pandemic situation at the time, on October 6, Pennsylvania Governor Tom Wolf announced increased crowd capacity limits for indoor and outdoor events including sporting events. These limits were based on venue size and whether the venue was indoors or outdoors. During the recruitment period and PA sessions, there were approximately 1000–2000 new COVID-19 cases being reported daily in central Pennsylvania. All study procedures reported here were approved by the Institutional Review Board of the investigators.

### 2.1 Intervention design

Given the growing body of evidence suggesting that people who perceive PA as enjoyable are more likely to both adopt and adhere to it (Lewis et al., 2015), this adult group sport play program, known as PlayFit, was specifically designed to maximize PA enjoyment (Sciamanna et al., 2017). The philosophy of PlayFit contrasts with most common opportunities for PA and sport among adults (e.g., fitness centers, recreational sports). Specifically, the philosophy of PlayFit is that perceptions of enjoyment during PA should supersede outcomes such as improving fitness, performance, and physical appearance. We theorize that when PA is enjoyable and, therefore, participants adhere to it, the secondary outcomes of fitness, performance, and appearance are likely to be realized despite not explicitly focusing on them.

PlayFit incorporates several tenets of self-determination theory (Ryan and Deci, 2000), hedonic theory (Ekkekakis and Dafermos, 2012), and the best-practices recommended in modern physical education (PE) (Vazou et al., 2019) to maximize PA enjoyment,improve automatic affective associations, and affective judgements (Zenko et al., 2016). For example, the games of PlayFit are made to be easier to play, reducing motor skillfulness barriers to entry and the likelihood of perceptions of motor skill incompetence and the unpleasant emotions associated with poor performances (e.g., embarrassment, shame). PlayFit also allows participants to self-pace and self-regulate their effort and intensity (i.e., promoting autonomy), and establishes a culture conducive to positive peer socialization (i.e., increasing feelings of relatedness) by deemphasizing interpersonal comparisons of skill and performance and allowing for frequent socialization breaks. We aim to provide an alternative form of PA for adults who may have interest in group sport and/or PA, but were discouraged by negative past experiences with traditional sport and PE (Cardinal et al., 2013) or are simply looking for a non-competitive option for sport participation. Prior to its indefinite suspension, PlayFit consisted of five modified sport games, including: ultimate Frisbee, ultimate football, handball, netball, and soccer. Because this pilot project was completed outdoors on grass and a basketball hoop was unavailable, the games played in this study were ultimate Frisbee, ultimate football, handball and soccer.

### 2.2 Participants

**Fig. 1:**
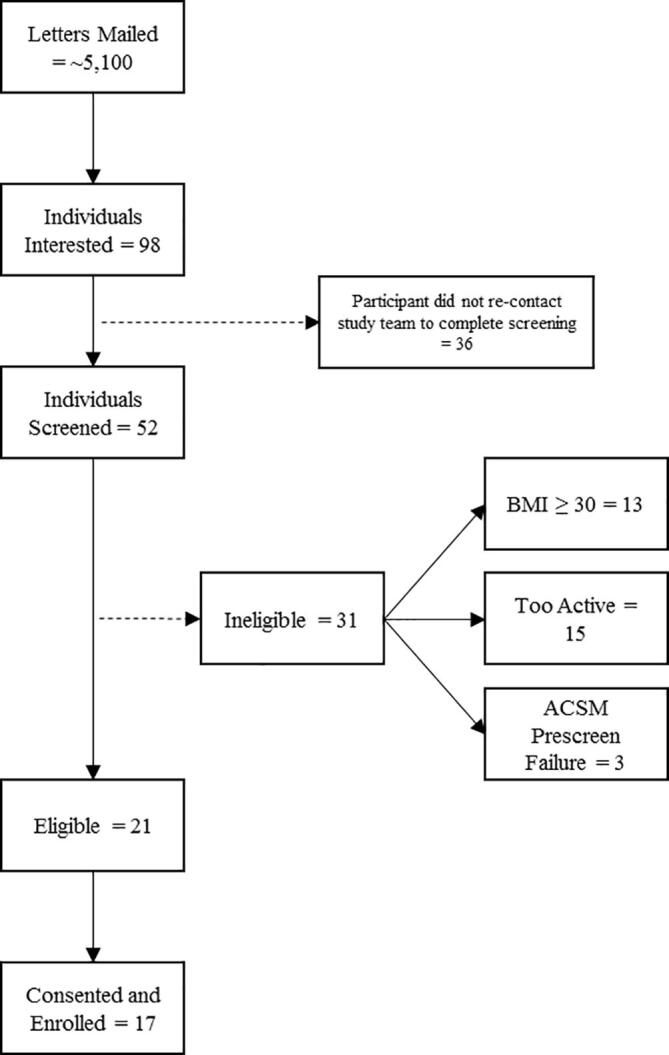
Study recruitment flowchart.

We recruited adults between 18 and 50 years of age, healthy enough for moderate-to vigorous-intensity physical activity (MVPA; i.e., answering “no” to all items of the ACSM Exercise Pre-Participation Health Screening Questionnaire for Exercise Professionals), sedentary (i.e., less than 90-minutes of MVPA per week), and not participating in another PA or weight loss program. Additionally, because of the emerging risk-factors associated with severe COVID-19 symptoms, we implemented additional exclusion criteria (Centers for Disease Control and Prevention, 2020). These included limiting the study to those under 50 years of age and those with a body mass index (BMI) less than 30 kg/m^2^ (i.e., those without obesity). During a condensed two-week recruitment period, we mailed approximately 5,100 letters to adults living in the same county as the research site. Of those who received letters, 98 (1.9%) responded with interest, and 52 were further screened for eligibility (see Fig. 1 for recruitment flowchart). The most common reasons for ineligibility were that the individual reported too much weekly MVPA (*n =* 15*)* or reported a BMI ≥ 30 (*n =* 13). Of the 21 eligible individuals, 17 (*n*_male_ = 7, *n*_female_ = 10, *mean*_age_ = 31.6, SD *=* 7.3, *mean*_BMI_ = 23.6, SD = 3.2) provided informed consent and enrolled in the study. This rate of interest in participating in the study was similar to the rates we observed for our parent trial that recruited prior to the COVID-19 pandemic, where, of approximately 21,000 letters mailed, 3% contacted our team expressing interest. However, given these more stringent eligibility criteria, it is inappropriate to compare actual rates of enrollment.

### 2.3 Measures

**Program attendance.** Attendance was recorded at each session by the group leader.

**Perceptions of the program.** During each debriefing phone conversation following the end of the study, the participants were asked several questions regarding their perceptions of the PA program and the COVID-19 safety modifications. Enjoyment was assessed using a single, face-valid item: “On a scale of 0–10, with 0 being no enjoyment and 10 being the highest amount of enjoyment: How much did you enjoy the exercise program?” We also used 0–10 scales to assess how likely each participant was to recommend the program to a friend, family member, or colleague (i.e., net-promoter score (Reichheld, 2003), how socially connected each participant felt with others, to what degree the individuals felt safe during the program, and whether they would continue the program, if it were offered again in the future. For each question, participants could further expound by providing qualitative responses.

**COVID-19 wellness outcomes.** Participants were each screened by phone on the morning of each PA session, upon arrival at the site, and 10-days following the final PA session for exposure to and existing or new symptoms of COVID-19. The screenings included whether the participant had been diagnosed with or had close contact with a person who tested positive for or was being tested for COVID-19 and reports of fever, dry cough, shortness of breath, sore throat, chills, aches, headache, and/or loss of taste or smell.

### 2.4 Procedure

Based on the best-practices recommended by amateur and professional sporting agencies (Sikka et al., 2021, League, 2020, League, 2020, Aschburner, 2020, Institute, 2020, Pennsylvania Interscholastic Athletic Association, 2020), we adhered to the following safety protocol: 1) all participants were required to wear masks at all times during PA and rest, 2) upon arrival, the temperature of each participant was assessed using a touchless thermometer, 3) participants were asked about new COVID-19 exposure and/or symptoms, 4) the participants were frequently reminded, to the best of their ability, to maintain at least six-feet between one another, 5) the sport implements (i.e., ball, Frisbee) were frequently sanitized by staff and the participants were to use hand sanitizer between play periods, and 6) participants were required to provide their own hydration.

The PlayFit sessions were held outdoors on a level, grass soccer field in central Pennsylvania during October 2020, from 5:30 pm-6:30 pm, on four weekday evenings with at least one day between sessions, over two consecutive weeks. Because PlayFit is designed to be easier to play, especially for individuals with low motor skillfulness and who are sedentary, overweight, and/or unfit, the field size was 180′ x 90′ and demarcated using cones. We used small (4′ x 6′) pop-up nets for soccer and handball. A slightly under-inflated volleyball (i.e., inflated to 2.0 lb per square inch) was used for soccer, to reduce the distance and speed at which it could travel along with making it lower impact if it struck the body. A soft, flexible Frisbee was used for ultimate Frisbee to reduce flight distances and difficulty with catching. For similar reasons, a foam football was used for ultimate football. Finally, a rubberized dodgeball was used for handball. The rulesets for each game were also minimized. For example, we did not keep score. Participants could stand and attempt to score from anywhere (i.e., no offside rule), drops or out-of-bounds were automatically turned-over to the other team, and there were no goaltenders. During ultimate Frisbee, football, and handball, a player could take up to 2-steps before passing to an open teammate using any type of pass or attempting to score. The players could take a break at any time by “switching out” with a teammate or could simply reduce their effort without repercussion. To avoid singling out individuals, when providing public feedback to participants, the session leader avoided specific comments (e.g., “*Great pass 'participant name!'*”), instead providing general comments to the entire group (e.g., “*Great effort everyone!*”). Individual positive feedback was reserved for private conversations. Celebrations between participants and the leader were limited to verbal interactions (i.e., no high-fives). Once the COVID-19 screening protocol was completed, a trained PlayFit leader paired participants to play five-minutes of “catch” using the sport implement of the day as a warm-up prior to gameplay. The participants were randomly assigned to teams each day and wore colored mesh jerseys to differentiate one another. During the 60-minute play session, individual periods were eight-minutes long, and each period was followed by a five-minute socialization, water, and sanitization break.

### 2.5 Analysis

Independent-samples t-tests were used to examine differences in perceptions of program enjoyment, social connectedness, safety, and whether participants would recommend the program to others by sex, age group (under or over 30 years of age), and BMI category (normal weight or overweight). In addition, we examined relationships between attendance rate and sex, age group, and BMI category using chi-squares tests. Because we conducted 15 tests of probability, our Bonferroni-corrected alpha was set to 0.003 (i.e., 0.05/15), to reduce the likelihood of Type I error.

## 3 Results

**COVID-19 symptoms and/or diagnoses.** No participants reported or were screened by research staff to have COVID-19 symptoms before, during, or after each of the PA sessions. In addition, no participants reported new COVID-19 symptoms or diagnoses during debriefing interviews 10-days following the final session.

**Program attendance.** The session attendance rate during the four sessions was 94%, 100%, 94%, and 76% (91% overall). The first three sessions were held in clear, sunny, weather and the final session was overcast and cooler with light rain. There were no significant relationships between program attendance and sex, age group, and BMI category.

**Program perception.** Overall, the participants reported a mean enjoyment score of 8.9/10 (*median* = 9.0, *range* = 8–10), a mean score on the likelihood to recommend the program to others of 9.4/10 (*median* = 10, *range* = 8–10), and all participants (*n* = 17) responded that they would be willing to continue the program if offered in the future. The mean score for perceptions of safety was 8.6/10 (*median* = 8, *range* = 7–10) and, despite the inherent limitations created by compulsory masking and social distancing, the mean score for perceptions of social connectedness to others was 7.9/10 (*median* = 8, *range* = 4–10). Finally, there were no significant differences in the mean scores on these variables based on sex, age, or BMI (all *p* > .003).

**Qualitative program perception.** A selection of program qualitative responses are presented in Table 2. Participants reportedly enjoyed the opportunity to be physically active outdoors and meet others after being sedentary and under stay-at-home orders during the pandemic. They also commented that PlayFit itself was low-stakes and non-competitive, with a supportive and friendly culture. The participants noted that wearing the masks during the PA periods did not negatively impact their experience, though it did make it more difficult to communicate at times. Some of the participants did respond that they missed being allowed to celebrate using physical means, such as “high-fives”, though the verbal encouragement from one another seemed to reduce the negative impact of this rule. Some participants suggested that they would have felt safer with sport games using no hands, but at the same time recognized that this would be unrealistic and that the frequent sanitization of the sport implements increased their perceptions of safety. All quantitative and qualitative data are included in Supplementary File 1.

**Table 2:** Participant qualitative themes and selected example statements.

**Theme**	**Example**
Program enjoyment “What did you enjoy about the program?”	• “I liked that it was a group of similar people (fitness-wise) so nobody was or felt out of place. I liked that it wasn't focused on rules or winning - you were just going to have fun. I also enjoyed the friendly level of competition in this.” Female – 23 years of age • “I'm used to playing everything competitively and it was really nice for a change to just get out and join a group of people who weren't as competitive. It made me focus more on the enjoyment as opposed to crushing opponents.” Female – 27 years of age
Program recommendation “Why would you recommend (or not recommend) the program?”	• “I'd recommend the program because I had a good time and think it's a program different types of people would enjoy. You don't need to be most alethic - or you could be, it really doesn't matter. This is universal, is for all, and many would enjoy.” Female – 23 years of age • “I know a lot of people who have a hard time getting out and about and moving - I think it'd be good for them, and something different, too, rather than just walking on a treadmill. Get people out for something different.” Male – 43 years of age
Safety perceptions “How did the focus on safety impact your overall program experience?”	• “The fact that we would sanitize the game ball and our hands after every round definitely helped with the safety. Also, the fact that we were required to wear our masks at all times helped, too.” Male – 31 years of age • “Only thing is wanting to celebrate and high-five with each other, and with the masks and sweating, you just unconsciously pull the mask down and wipe the sweat off of your face. In my opinion, you just need to be more conscious of those things - don't high-five, and don't wipe your face.” Female 39 – years of age
Social connectedness “How did the focus on safety impact your overall sense of social connectedness?”	• “The focus was a positive because the rules were established and made everyone feel safe and know they/we can still talk and converse at a distance. Again, I thought it was very positive.” Male – 43 years of age • “Net positive - I'm honing in on shared experience of all of us sanitizing at the end of each period, and I think this increased group cohesion. The promoting of the safety feature as taking part in the program helped to bring us together.” – Male 35 years of age
Willingness to continue “What aspects of the program made you feel this way?”	• YES: “I liked a scheduled regimen for my to-do list. Even on the rainy night, I knew the program was there, so I went. I was glad I went because I ended up having fun.” Female – 23 years of age • YES: “I enjoyed the social part and the fact that it was not boring. It was sports that we could all participate in regardless of our skill level. This wasn't just recess playing dodgeball.” Female – 40 years of age

## 4 Discussion

The purpose of this study was to examine whether an adult outdoor group sport play program designed to maximize enjoyment that incorporated COVID-19 risk mitigation strategies was feasible, acceptable, and well-adhered to, while remaining enjoyable. Our results suggest that the program was feasible, well-accepted, and likely to be adhered to over the short-term among healthy, but sedentary adults, albeit those without obesity. Despite enforcing safety precautions, such as universal masking and social distancing, the participants, in general, reported enjoying the program, as well as experiencing feelings of social connectedness with the other participants. In addition, the overall attendance rate was 91% and no participants reported new COVID-19 symptoms or positive test results during the two-weeks of activity sessions and over the 10-days following the final PA program session. While not as overwhelming as post-Thanksgiving (i.e., late November) 2020, COVID-19 was circulating actively at that time in the Pennsylvania community where the PA program sessions were held (i.e., around 1000–2000 new cases diagnosed daily).

Although these results may be of interest to PA and sport professionals and researchers, several limitations must be considered. First, this study was conducted in October 2020, following nearly eight-months of COVID-19 pandemic-related restrictions in the U.S. Several participants may have strongly desired to engage with *any* program that allowed for socialization because of the negative social and mental health impacts of mandatory stay-at-home orders and quarantine requirements. Indeed, several participants expressed their happiness with having the opportunity to socialize during the pandemic, without specifically alluding to PA. These individuals may not have been as motivated to join this specific PA program if other options were available in a non-pandemic setting. In addition, the PA sessions were conducted outdoors and safe group PA programming may be more difficult and sometimes infeasible indoors, as the poor ventilation often present indoors could be attributed to increased spread of COVID-19 (World Health Organization, 2009, American Society of Heating Refrigerating and Air-Conditioning Engineers et al., 2008). At the same time, several indoor professional, amateur, and youth sport leagues have held regular events with little evidence of increased spread of the virus among players (Sikka et al., 2021, League, 2020, League, 2020, Aschburner, 2020, Institute, 2020, Pennsylvania Interscholastic Athletic Association, 2020). The generalizability of the present study may also be reduced because the sessions were held outdoors in mild fall (i.e., ~50°F, 10 °C), but not winter temperatures, when outdoor PA rates tend to be lower (Turrisi et al., 2021). Additionally, if the study sessions were held during the summer with high heat and high humidity, the comfort level of wearing a mask during MVPA expressed by the participants may have changed. Finally, the sedentary participants in the current study were restricted to those who were normal weight to overweight and their experiences may not be the same as those with obesity, who sometimes experience even moderate-intensity PA as aversive (Ekkekakis and Lind, 2006).

Our results suggest that outdoor PA play among sedentary adults during the COVID-19, with health precautions in place, was feasible, acceptable, and well-adhered to despite the burdens associated with added health precautions. These results may have implications for possible future pandemics with viral transmission similar to COVID-19 (i.e., mostly airbornee transmission via respiratory droplets), as some researchers suggest the likelihood of future pandemics is growing due to increased globalization, urbanization, climate change, human-animal contact, and health care worker shortages (Gibb et al., 2020).

## CRediT authorship contribution statement

**Matthew A. Ladwig:** Conceptualization, Methodology, Data curation, Writing - original draft, Investigation, Supervision, Formal analysis. **Christopher N. Sciamanna:** Conceptualization, Methodology, Writing - review & editing, Supervision. **Kayla Rutt:** Methodology, Investigation, Writing - review & editing. **Joshua Blaker:** Methodology, Investigation, Writing - review & editing. **Kalen Kearcher:** Methodology, Investigation, Writing - review & editing. **Brandon J. Auer:** Methodology, Writing - review & editing. **Liza S. Rovniak:** Methodology, Writing - review & editing. **David E. Conroy:** Methodology, Writing - review & editing. **Jinger S. Gottschall:** Methodology, Writing - review & editing. **Matthew L. Silvis:** Methodology, Supervision, Writing - review & editing. **Joshua M. Smyth:** Methodology, Writing - review & editing. **Ming Wang:** Methodology, Writing - review & editing.

## Declaration of Competing Interest

The authors declare the following financial interests/personal relationships which may be considered: Christopher Sciamanna has an investment, such as stock, in a company which has begun to investigate the possibility of creating a business that provides exercise programs.
